# Supplementary material for: Migratory orientation in a narrow avian hybrid zone
Source: PeerJ. 2017 Apr 18;5:e3201. doi: 10.7717/peerj.3201 (PMC5398278; doi:10.7717/peerj.3201)
Supplement: Supplemental Information 1 [file peerj-05-3201-s001.docx]

**SUPPLEMENTARY INFORMATION**

**Table S1 –** radR parameter settings. Parameters in bold were varied between light environments.

| **radR parameter** | **Sun directly on Cage** | **Sunset (twilight)** | **Darkness** |
| --- | --- | --- | --- |
| Old stats weighting | 0.95 | 0.95 | 0.95 |
| Hot score threshold (high) | **200** | **200** | **10** |
| Hot score threshold (low) | **-10** | **-10** | **-10** |
| Samples per cell | 4 | 4 | 4 |
| Pulses per cell | 4 | 4 | 4 |
| Min blip samples | 100 | 100 | 100 |
| Max blip samples | -1 | -1 | -1 |
| Min Blip area | 300 | 300 | 300 |
| Min Blip area | -2 | -2 | -2 |
| Min angular span | 2 | 2 | 2 |
| Max angular span | -1 | -1 | -1 |
| Min radial span | 1 | 1 | 1 |
| Max radial span | -1 | -1 | -1 |
| Filter by logical expression | **Int < 0.37** & range < 10000 | **Int < 0.5** & range < 10000 | **Int >0.6** & range < 10000 |

**Figure S1** – An example of radR output and analysis for one individual (“975”). Each point represents a single object identified by radR during the orientation trial. The central white area represents the circular perch, which is excluded from the analysis. The red crosshairs illustrates the average plus the SD of all XY points. For the final analysis each of the points is converted to an angle, from which the mean orientation and concentration is calculated.

**Figure S2 –** The same individual, this time showing variation in behavioural traits measured over the trial. Rho, or concentration of points, is shown in the left panel split into different time segments relative to sunset. The right panel shows the angles (average +/- SD) for each of these time segments, illustrating a consistent orientation tendency for this individual.

**Figure S3 –** Percentage of checklists between 1990-2014 in Alberta that reported yellow-rumped warblers during the fall migratory period (August-November). Data provided by [www.ebird.com](http://www.ebird.com).
